# Pharmacokinetics and pharmacodynamics across infusion rates of intravenously administered nipocalimab: results of a phase 1, placebo-controlled study

**DOI:** 10.3389/fnins.2024.1302714

**Published:** 2024-02-01

**Authors:** Jocelyn H. Leu, An Vermeulen, Claudia Abbes, Santiago Arroyo, William S. Denney, Leona E. Ling

**Affiliations:** ^1^Janssen Research & Development, LLC, Spring House, PA, United States; ^2^Janssen Research & Development, LLC, a Division of Janssen Pharmaceutica NV, Beerse, Belgium; ^3^Momenta Pharmaceuticals, Inc., Cambridge, MA, United States; ^4^Human Predictions, LLC, Boston, MA, United States; ^5^Janssen Research & Development, LLC, Cambridge, MA, United States

**Keywords:** neonatal Fc receptor, monoclonal antibodies, phase 1 clinical trial, intravenous infusions, pharmacokinetics, immunoglobulin G

## Abstract

**Introduction:**

Nipocalimab is a high-affinity, fully human, aglycosylated, effectorless, immunoglobulin G (IgG) 1 monoclonal antibody that targets the neonatal Fc receptor (FcRn), decreases systemic IgG including autoantibodies, and is under development in several IgG autoantibody- and alloantibody-mediated diseases, including generalized myasthenia gravis, chronic inflammatory demyelinating polyneuropathy, maternal-fetal medicine, and multiple other therapeutic areas. An initial phase 1 study with single and multiple ascending doses of nipocalimab infused intravenously (IV) over 2 h demonstrated dose-dependent serum pharmacokinetics and IgG reductions, with an adverse event (AE) profile comparable to placebo.

**Methods:**

The current investigation evaluates the safety, tolerability, pharmacokinetics, and pharmacodynamics of single doses of nipocalimab across various IV infusion rates in a randomized, double-blind, placebo-controlled, sequential-dose study. Forty participants were randomized to receive nipocalimab 30 mg/kg over 60, 30, 15 or 7.5 min (0.5, 1, 2, or 4 mg/kg/min); nipocalimab 60 mg/kg over 15 min (4 mg/kg/min); or matching placebo.

**Results:**

At doses up to 60 mg/kg and infusion rates up to 4 mg/kg/min (maximum clinically feasible rate), single doses of nipocalimab were tolerable, with 12 (40%) participants experiencing AEs across nipocalimab cohorts compared with 1 (10%) participant in the placebo cohort. AEs deemed treatment related occurred in 6 (20%) participants receiving nipocalimab and 1 (10%) participant receiving placebo. None of the AEs were severe, and no participants discontinued treatment due to AEs. Nipocalimab provided consistent, dose-dependent serum pharmacokinetics and IgG reductions, regardless of infusion rate.

**Discussion:**

This study supports the use of shortened durations of nipocalimab infusion for future studies.

## Graphical abstract

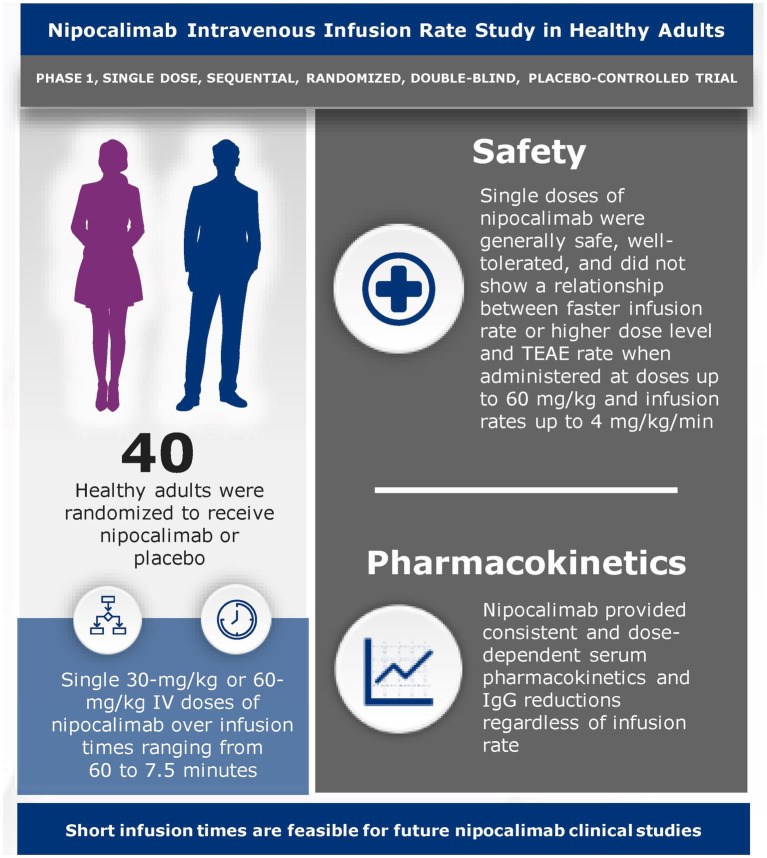

## 1 Introduction

Autoantibody-mediated diseases impact nearly 3% of the global population and comprise over 80 chronic autoimmune and acute alloimmune conditions across multiple therapeutic areas (Eggert et al., 2010; Ludwig et al., 2017). Traditional therapeutic strategies for the treatment of chronic autoantibody-mediated diseases include conventional immunosuppression, the use of B-cell–targeting drugs to reduce antibody production, the removal of monomeric pathogenic autoantibodies and immune complexes via plasma exchange (PLEX), immunoadsorption (IA), or intravenous immunoglobulin (IVIG) (Shock et al., 2020). However, these treatment options may lack adequate efficacy, be nonspecific (resulting in substantial safety and/or tolerability issues with long-term exposure), and impose a substantial burden to patients and health care systems (Osanan et al., 2012; Strengers and Klein, 2016; Bacci et al., 2019; US Food and Drug Administration, 2019; Jacob et al., 2021). There is a significant unmet need for safe and targeted therapies to address the underlying cause of auto- and alloantibody-mediated diseases.

The neonatal Fc receptor (FcRn) is a promising treatment target for immunoglobulin G (IgG) antibody–mediated diseases under evaluation in multiple therapeutic areas, including neuroimmunology, benign hematology, rheumatology, and maternal fetal medicine (Gable and Guptill, 2019; Chalayer et al., 2022; Pyzik et al., 2023). FcRn is responsible for the long 21-day half-life of IgG as it specifically binds and recycles IgG and albumin preventing their clearance through the intracellular lysosomal degradation pathway (Roopenian and Akilesh, 2007). Targeted blockade of FcRn-IgG binding inhibits IgG recycling and accelerates the destruction of pathogenic and total IgG. Lowering IgG (including pathogenic IgG) could provide significant therapeutic benefit for patients with IgG auto/alloantibody-mediated diseases (Pyzik et al., 2023). Determining the optimal administration and use of FcRn-targeted agents will be important in addressing the underlying cause of auto- and alloantibody-mediated diseases to reduce disease burden on patients and health care systems.

Efgartigimod and rozanolixizumab were the first two FcRn-targeted agents approved by the US Food and Drug Administration for the treatment of generalized myasthenia gravis (gMG), the chronic neuromuscular disease in which the role of pathogenic IgG is well established (Gilhus, 2016; Howard et al., 2019; Bril et al., 2021; Howard et al., 2021; Bril et al., 2023).

Nipocalimab is a high-affinity, fully human, aglycosylated, effectorless, IgG1 monoclonal antibody designed to selectively block the IgG binding site on FcRn to increase IgG clearance and reduce IgG auto/alloantibody-mediated disease pathology. Unlike other FcRn-targeted agents, nipocalimab binds with picomolar affinity to FcRn at both the endosomal and extracellular pH of 6.0 and 7.4 allowing rapid full occupancy of FcRn throughout the recycling pathway. These attributes result in the potential for rapid, deep and sustained effects on pathogenic IgG and disease response (Ling et al., 2019; Antozzi et al., 2024). While nipocalimab selectively and effectively increases IgG clearance, it spares IgG production and other key humoral and cellular immune functions (Ling et al., 2022).

The safety and efficacy of nipocalimab are being evaluated across a broad range of IgG auto- and alloantibody-mediated diseases, including diseases in neurology, dermatology, rheumatology, immunohematology, and alloantibody-mediated maternal fetal medicine indications, many of which are rare with limited treatment options (ClinicalTrials.gov, 2019a,b, 2021a,b,c,d,e, 2022a,b) In a phase 2 study of patients with gMG (Vivacity-MG [*N* = 67]; ClinicalTrials.gov Identifier: NCT03772587), nipocalimab treatment resulted in substantial, rapid, and dose-dependent reductions in total serum IgG and in anti-acetylcholine receptor–specific autoantibodies compared with placebo (Antozzi et al., 2024). Myasthenia Gravis Activities of Daily Living scores significantly improved with nipocalimab compared with placebo, while the frequency of adverse events (AEs) was comparable to placebo.

Nipocalimab was well tolerated, with an acceptable safety profile, and exhibited clear dose, pharmacokinetic (PK), and pharmacodynamic (PD) relationships when administered as an infusion over 2 h at rates up to 0.5 mg/kg/min in a first-in-human, single and multiple ascending dose study in healthy participants (Ling et al., 2019). In subsequent studies and in clinical practice, shorter durations of infusion are desirable if tolerability and safety can be maintained and PK and PD remain consistent. The study presented here aimed to assess the safety, tolerability, PK, and PD of single 30 or 60-mg/kg intravenous (IV) doses of nipocalimab in healthy adults over infusion times ranging from 60 to 7.5 min to examine shorter nipocalimab IV infusions for potential use in clinical studies and in clinical practice.

## 2 Materials and methods

### 2.1 Ethics

This study was in compliance with the International Council for Harmonisation Harmonised Tripartite Guideline regarding good clinical practice. Study protocols were reviewed by the Advarra, Inc. institutional review board and is registered with the US Food and Drug Administration (Registration Number: IRB 00000971). The study was conducted in accordance with the Declaration of Helsinki and was consistent with good clinical practices and regulatory requirements. Participants provided written informed consent prior to enrollment.

### 2.2 Participants

Eligible participants were males or females aged 18 to 55 years with no clinically significant medical conditions, body mass index (BMI) ≥18.5 and < 32.0 kg/m^2^, body weight ≥ 50 and ≤ 110 kg, and total serum IgG levels ≥1,000 mg/dL at screening. Women of childbearing potential and nonsterile men agreed to use effective contraception to prevent pregnancy prior to dosing, during the study, or in a specified period after dosing. Participants with a history of disease(s) of the immune system, use of immunosuppressive therapy, history or presence of hypersensitivity or idiosyncratic reaction to the study drug or related compounds, or history or presence of a clinically significant medical or psychiatric condition were excluded.

### 2.3 Study design

**Figure 1:**
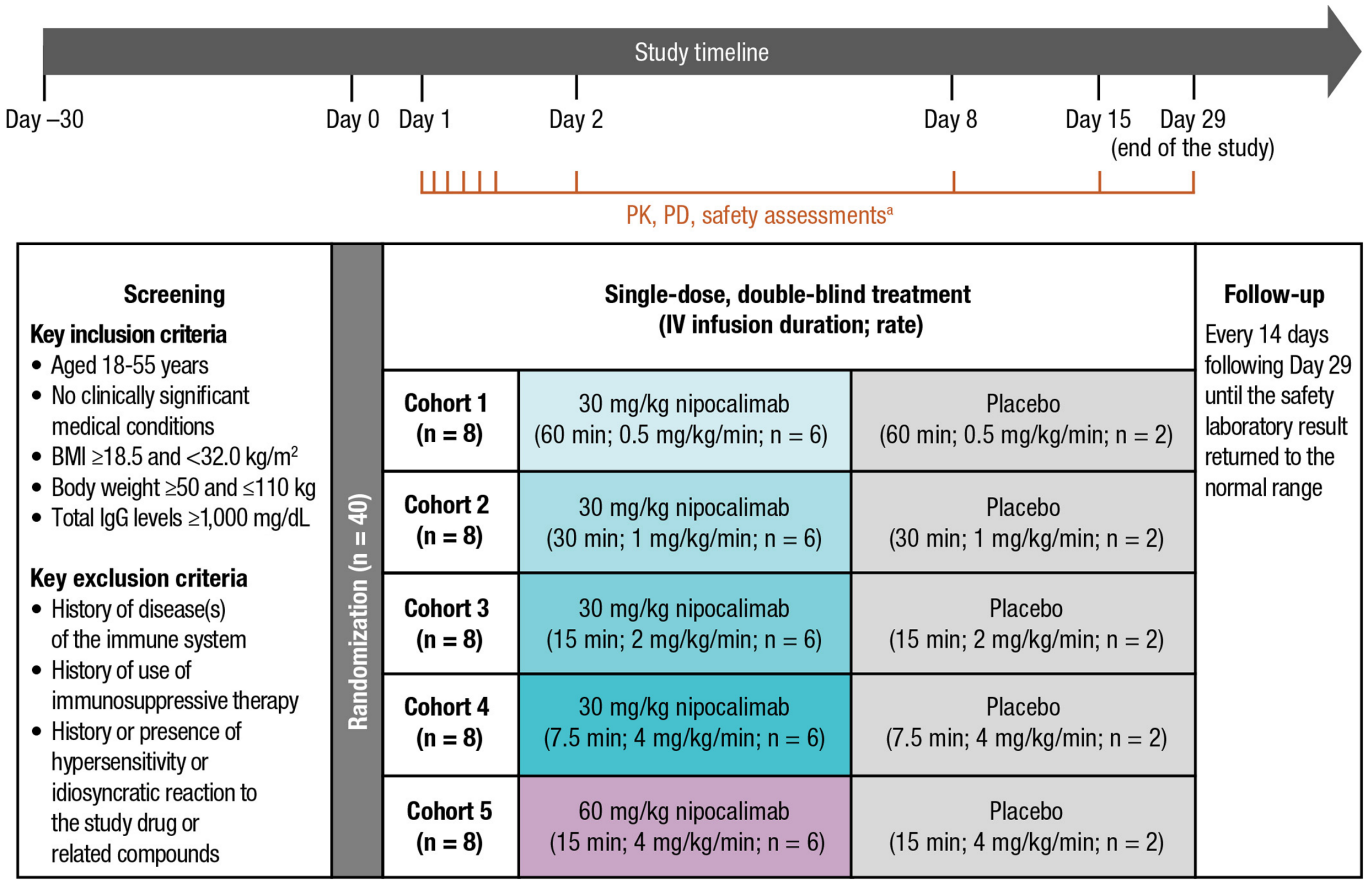
Study design of a phase 1, single-dose, sequential escalating-dose, randomized, double-blind, placebo-controlled, infusion rate study. BMI, body mass index; EOI, end of infusion; IgG, immunoglobulin G; IV, intravenous; MOI, middle of infusion; PD, pharmacodynamic; PK, pharmacokinetic. ^a^The PK assessment time points included Day 1 predose, MOI, EOI, EOI + 0.083 h, EOI + 0.25 h, EOI + 1 h; Day 2 (24 h postdose); Day 8 (169 h postdose); and Day 15 (337 h post dose). The PD and safety assessment time points included Day −1, Day 2 (24 h postdose), Day 8 (169 h postdose), Day 15 (337 h postdose), and Day 29 (672 h postdose), with an optional test every 14 days post−Day 29 until clinically significant laboratory test results returned to normal ranges.

This single-infusion, sequential, randomized, double-blind, placebo-controlled, escalating dose and infusion rate, phase 1 study was conducted at a single clinical site in the United States. The study consisted of a screening phase (within 30 days before study drug administration), a 29-day study phase (single IV doses of nipocalimab were given on Day 1, PK parameters were assessed for 336 h [up to Day 15], and PD parameters were assessed for 672 h [up to Day 29]), and a follow-up phase (every 14 days following Day 29 until safety laboratory results returned to the normal range; Figure 1). Safety parameters were assessed throughout the study, including the screening phase; Day −1; prespecified time points between Day 1 and Day 2, Day 8, Day 15, and Day 29; and the follow-up phase. On Day 1, participants were randomized to 1 of 5 cohorts (*n* = 8 per cohort [nipocalimab, *n* = 6; placebo, *n* = 2]) to receive nipocalimab 30 mg/kg IV infused over 60, 30, 15, or 7.5 min (0.5, 1, 2, or 4 mg/kg/min); nipocalimab 60 mg/kg IV infused over 15 min (4 mg/kg/min); or matching placebo. The maximum infusion rate of 4 mg/kg/min was the fastest rate that was feasible at the clinical site. Escalation to the next dose level was permitted following review by a safety monitoring committee.

### 2.4 Safety and tolerability evaluations

Safety and tolerability of study treatments were assessed via laboratory tests, vital signs, electrocardiogram (ECG), and by the frequency and nature of treatment-emergent AEs (TEAEs) and serious AEs (SAEs). AEs were coded using the *Medical Dictionary for Regulatory Activities*, version 21.1. Optional laboratory tests were performed every 14 days post–Day 29 until safety laboratory test results returned to the normal range.

### 2.5 PK evaluations

Blood samples for PK analysis of nipocalimab were collected at Day 1 predose and at prespecified time points between Day 1 and Day 2, Day 8, and Day 15. PK samples at the middle and end of infusion varied for each cohort as a result of different infusion rates. Serum concentrations of nipocalimab were determined using an enzyme-linked immunosorbent assay method validated with respect to accuracy, precision, linearity, sensitivity, and specificity (Charles River Laboratories, Reno, NV, USA). The limits of quantification for nipocalimab were 0.150 to 1,250 μg/mL. Standard serum PK parameters were derived including maximum observed serum concentration (C_max_), time to reach the maximum observed serum concentration (T_max_), area under the serum concentration-time curve (AUC) from time 0 (start of infusion) to the time of the last observed nonzero concentration (AUC_0-last_), AUC from time 0 (start of infusion) extrapolated to infinity (AUC_0-inf_), half-life (t_1/2_), observed concentration at the end of infusion (C_eoi_), clearance (CL), and volume of distribution (Vz).

### 2.6 PD evaluations

Blood samples for PD analysis of total serum IgG and albumin concentrations were collected at Day −1, Day 2, Day 8 ± 1, Day 15 ± 2, and Day 29 ± 3, with an optional test every 14 days post−Day 29 until clinically significant laboratory test results returned to normal ranges (ie, 768–1,632 mg/dL for total serum IgG). Total serum IgG and albumin concentrations were analyzed by Celerion (Tempe, AZ, USA). The proportion of participants with hypoalbuminemia categorized based on Common Terminology Criteria for Adverse Events (grade 3: <20 g/L, grade 2: 20 to <30 g/L, grade 1: 30 to <40 g/L) or with normal (40–50 g/L) or high albumin levels (>50 g/L) was also reported at each postdose time point compared with baseline.

### 2.7 Statistical analysis

Descriptive statistics were used for participant characteristics, serum concentrations of nipocalimab and total IgG, derived PK parameters (as appropriate), laboratory tests, ECG, and vital signs. PK parameters were calculated from the serum nipocalimab concentration-time data using noncompartmental analysis (Phoenix® WinNonlin®, version 7.0; Certara, Princeton, NJ, USA). All statistical analysis and reporting were generated using SAS for Windows, version 9.4 (SAS Institute, Cary, NC, USA).

## 3 Results

### 3.1 Participants

A total of 40 participants received study treatment and were included in the safety analysis. Of those, 30 participants received nipocalimab and were included in the PK and PD analyses. A total of 39 participants completed the study, and 1 participant who received placebo was considered lost to follow-up after missing a scheduled return visit (14 days after end of study). Baseline demographic and selected clinical characteristics were similar across all treatment cohorts (Table 1). The mean age was 37.0 years, 70% were female, 92.5% were White, mean baseline BMI was 26.8 kg/m^2^, and mean baseline serum IgG was 1,183.9 mg/dL.

**Table 1:** Demographic and other baseline characteristics.

Characteristic^a^	Nipocalimab						Placebo (*n* = 10)
	30 mg/kg (60 min; 0.5 mg/kg/min) (*n* = 6)	30 mg/kg (30 min; 1 mg/kg/min) (*n* = 6)	30 mg/kg (15 min; 2 mg/kg/min) (*n* = 6)	30 mg/kg (7.5 min; 4 mg/kg/min) (*n* = 6)	60 mg/kg (15 min; 4 mg/kg/min) (*n* = 6)	Total (*n* = 30)	
Sex, *n* (%)							
Female	4 (67)	3 (50)	5 (83)	5 (83)	5 (83)	22 (73)	6 (60)
Male	2 (33)	3 (50)	1 (17)	1 (17)	1 (17)	8 (27)	4 (40)
Ethnicity, *n* (%)							
Hispanic or Latino	5 (83)	4 (67)	5 (83)	5 (83)	6 (100)	25 (83)	7 (70)
Not Hispanic or Latino	1 (17)	2 (33)	1 (17)	1 (17)	0	5 (17)	3 (30)
Race, *n* (%)							
Black or African American	0	1 (17)	0	0	0	1 (3)	0
Native Hawaiian or other Pacific Islander	0	1 (17)	0	0	0	1 (3)	0
White	6 (100)	4 (67)	6 (100)	6 (100)	6 (100)	28 (93)	9 (90)
Mixed White, Black, or African American	0	0	0	0	0	0	1 (10)
Age, y	37.7 (6.5)	35.0 (8.7)	43.8 (11.0)	36.7 (11.7)	34.0 (11.0)	37.4 (9.9)	35.8 (10.9)
Weight, kg	75.2 (10.2)	77.2 (14.0)	71.5 (9.7)	74.1 (5.0)	73.9 (10.5)	74.4 (9.7)	71.5 (15.2)
Height, cm	167.2 (7.2)	169.8 (12.4)	161.8 (6.6)	162.7 (4.4)	164.5 (9.3)	165.2 (8.4)	167.1 (10.2)
BMI, kg/m^2^	26.9 (3.9)	26.7 (3.0)	27.3 (3.0)	28.1 (2.4)	27.3 (3.0)	27.2 (2.9)	25.4 (3.6)
Albumin, g/L	44.7 (1.8)	45.2 (2.8)	46.7 (2.9)	43.5 (3.3)	45.7 (2.0)	45.1 (2.7)	46.5 (1.8)
IgG, mg/dL	1,104.5 (261.5)	1,134.5 (209.7)	1,261.7 (173.9)	1,136.5 (200.9)	1,270.0 (174.8)	1,181.4 (204.6)	1,191.3 (208.4)
IgM, mg/dL	79.8 (25.5)	146.5 (27.6)	89.7 (49.5)	154.2 (91.9)	143.3 (78.2)	122.7 (64.8)	123.5 (39.7)
IgA, mg/dL	168.8 (95.2)	254.0 (72.2)	213.2 (63.9)	245.8 (91.1)	240.7 (46.8)	224.5 (77.3)	239.9 (83.6)
IgE, mg/dL	60.5 (114.2)	25.2 (31.2)	105.7 (131.1)	115.8 (128.3)	86.8 (159.0)	78.8 (117.0)	394.5 (1162.4)

BMI, body mass index; Ig, immunoglobulin; SD, standard deviation.

^a^Data are mean (SD) unless otherwise noted.

### 3.2 Safety

A total of 12 (40%) participants experienced TEAEs across all nipocalimab dosing cohorts, and 1 (10%) participant experienced TEAEs following the administration of placebo (Table 2). None of the TEAEs were severe, and there were no deaths, SAEs, or discontinuations of treatment due to TEAEs at any infusion rate of nipocalimab. A total of 6 (20%) participants in the nipocalimab dosing cohorts experienced TEAEs that were considered related to the study drug. All of these TEAEs were of mild (grade 1) or moderate (grade 2) severity and were observed to resolve by the end of the study, except for 3 grade 1 TEAEs in 1 participant in the 60-mg/kg cohort (15-min infusion duration; 4 mg/kg/min) who was lost to follow-up. Grade 2 infusion-site reactions were reported in 2 participants. Both were considered unrelated to the study drug. One participant in the 30-mg/kg cohort (60-min infusion duration; 0.5 mg/kg/min) exhibited a vessel puncture−site reaction on the right hand that began approximately 29 min following the start of the infusion and later developed ecchymosis, erythema, pain, redness, and swelling in the right hand. The AE was completely resolved after approximately 21 days. Another participant in the 30-mg/kg cohort (15-min infusion duration; 2 mg/kg/min) exhibited infusion-site discomfort at the right antecubital IV site without erythema, induration, or swelling that began 5 min following the start of the infusion. The AE resolved approximately 5 h after onset.

**Table 2:** Number of participants reporting TEAEs and most frequent TEAEs (in ≥2 participants) by dosing cohort.

TEAE, *n* (%)	Nipocalimab						Placebo (*n* = 10)
	30 mg/kg (60 min; 0.5 mg/kg/min) (*n* = 6)	30 mg/kg (30 min; 1 mg/kg/min) (*n* = 6)	30 mg/kg (15 min; 2 mg/kg/min) (*n* = 6)	30 mg/kg (7.5 min; 4 mg/kg/min) (*n* = 6)	60 mg/kg (15 min; 4 mg/kg/min) (*n* = 6)	Total (*n* = 30)	
Any TEAE	3 (50)	1 (17)	2 (33)	3 (50)	3 (50)	12 (40)	1 (10)
Death	0	0	0	0	0	0	0
Any serious TEAE	0	0	0	0	0	0	0
Any TEAE leading to discontinuation of study drug	0	0	0	0	0	0	0
Treatment-related adverse events	1 (17)	1 (17)	0	2 (33)	2 (33)	6 (20)	1 (10)
Any infusion-site reaction	1 (17)^a^	0	1 (17)^a^	0	0	2 (7)	0
Most frequent TEAE							
Headache	1 (17)^b^	1 (17)^b^	0	2 (33)^a^	2 (33)^b^	6 (20)	1 (10)^b^
Nausea	0	0	0	1 (17)^a^	2 (33)^b^	3 (10)	0
Vomiting	0	0	0	0	2 (33)	2 (7)	0
Back pain	0	0	1 (17)^a^	0	1 (17)^b^	2 (7)	0
Nasal congestion	0	0	0	2 (33)^a,b^	0	2 (7)	0
Rhinorrhea	0	0	0	2 (33)^b^	0	2 (7)	0
Pruritis	0	0	0	1 (17)^b^	1 (17)^b^	2 (7)	0
Rash	0	0	0	1 (17)^b^	1 (17)^b^	2 (7)	0

TEAE, treatment-emergent adverse event.

^a^Event reported by participant(s) considered moderate in intensity (grade 2).

^b^Event reported by participant(s) considered mild in intensity (grade 1).

The most frequently reported TEAE was headache, reported by 6 participants (20%) with nipocalimab and 1 participant (10%) with placebo. None of the headache AEs occurred during the infusion. Grade 1 headaches were reported by 4 participants receiving nipocalimab (1 each in the 30-mg/kg cohorts [60-min infusion duration, 0.5 mg/kg/min; 30-min infusion duration, 1 mg/kg/min] and 2 in the 60-mg/kg cohort [15-min infusion duration, 4 mg/kg/min]) and by 1 participant receiving placebo. Grade 2 headaches were reported by 2 participants receiving nipocalimab at 30 mg/kg (7.5-min infusion duration; 4 mg/kg/min). All headache AEs were considered related to the study treatment, except for 1 participant in the 60-mg/kg cohort (15-min infusion duration; 4 mg/kg/min). Grade 1 nausea was reported by 2 participants (6.7%) receiving nipocalimab (60-mg/kg cohort [15-min infusion duration; 4 mg/kg/min]). Grade 2 intermittent nausea occurred in 1 participant 4 h after the infusion of 30 mg/kg (7.5-min infusion duration; 4 mg/kg/min), lasted until Day 28, and was considered related to the study drug. Grade 1 nausea accompanied by grade 1 intermittent vomiting was reported in 2 participants receiving the 60-mg/kg infusion (15-min infusion duration; 4 mg/kg/min), occurred within 24 to 28 days after the infusion of the study drug, and was considered related to the study drug in 1 participant.

A total of 4 participants (3 receiving nipocalimab and 1 receiving placebo) experienced AEs that occurred within 24 h after the infusion and were considered related to the treatment. These AEs were mostly headaches, which began from 5 to 11 h after the beginning of the infusion. Also reported were numbness at the top of the head at 10 h after infusion by 1 participant (30 mg/kg cohort [30-min infusion duration; 1.0 mg/kg/min]), as well as nausea at 4 h after infusion and “arms feel cool” during the infusion by another participant (30 mg/kg cohort [7.5-min infusion duration; 4 mg/kg/min]). All resolved and did not require pausing or discontinuation of dosing.

There were no laboratory-related AEs during this study. All individual abnormal laboratory results were considered clinically insignificant and not related to the study drug. There were no clinically significant changes in ECG, vital signs, or physical exams.

### 3.3 PK

**Figure 2:**
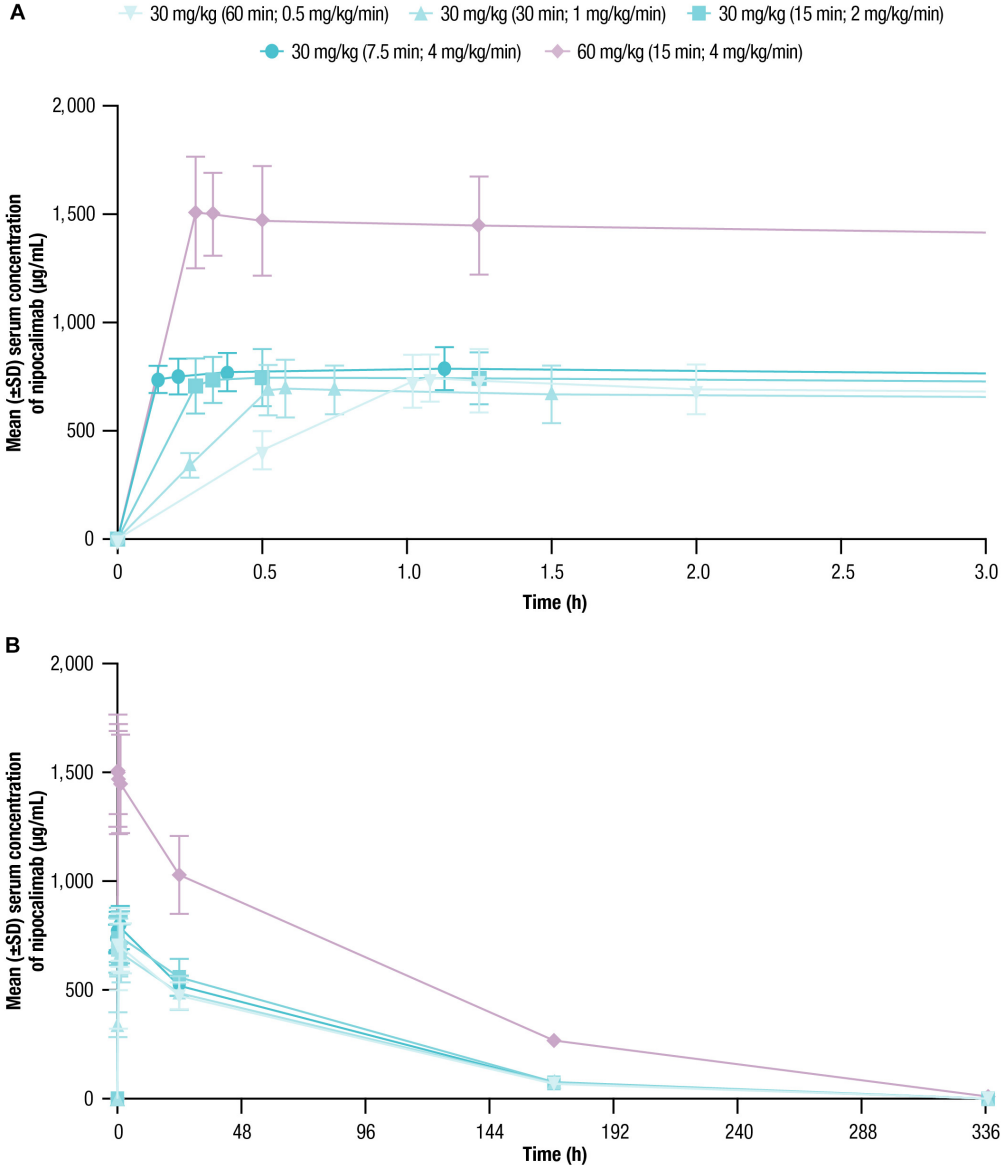
Serum nipocalimab concentrations over time were assessed for up to 336 h following single doses of nipocalimab (30 and 60 mg/kg) administered at escalating infusion rates (0.5–4 mg/kg/min). **(A)** Mean (±SD) serum nipocalimab concentrations over the first 3 h postdose (*n* = 6). **(B)** Mean (±SD) serum nipocalimab concentrations over the entire period of 336 h postdose (*n* = 6). SD, standard deviation.

The mean serum nipocalimab concentration-time profiles were similar following all 30-mg/kg infusions regardless of infusion rate and were approximately 2 times higher following a 60-mg/kg infusion (Figure 2). In all treatment cohorts, mean serum concentrations of nipocalimab declined in a dose-dependent nonlinear manner, suggesting target-mediated drug disposition, and remained detectable for at least 169 h postdose for all 30-mg/kg cohorts and 337 h postdose for the 60-mg/kg cohort.

The geometric means of AUC_0-inf_ and C_max_ of nipocalimab in serum were unaffected by infusion rate when administered at 30 mg/kg (Table 3). Following a 60-mg/kg infusion, the AUCs were 2.4 to 2.8 times higher and C_max_ was 2.0 to 2.2 times higher than with the 30-mg/kg infusions. T_max_ following 30 and 60-mg/kg infusions ranged from 20 to 68 min after initiation of IV infusion. Higher infusion rates were associated with a numerical trend toward shorter T_max_, although a delayed T_max_ was observed at the highest infusion rate (30 mg/kg cohort [7.5-min infusion duration; 4 mg/kg/min]). Mean t_1/2_, CL, and Vz values ranged from 48.8 to 58.0 h, 0.03 to 0.05 L/h, and 2.6 to 3.4 L, respectively, across nipocalimab dosing cohorts. Numerical trends toward slower CL and lower Vz were observed with shorter infusion following 30-mg/kg infusions, except for in the 4-mg/kg/min cohort (*n* = 1).

**Table 3:** Serum PK parameters of nipocalimab.

PK parameter^a^	30 mg/kg (60 min; 0.5 mg/kg/min)	30 mg/kg (30 min; 1 mg/kg/min)	30 mg/kg (15 min; 2 mg/kg/min)	30 mg/kg (7.5 min; 4 mg/kg/min)	60 mg/kg (15 min; 4 mg/kg/min)
AUC_0-last_, μg∙h/mL	43,050 (22.4) [*n* = 6]	43,530 (28.2) [*n* = 6]	49,950 (19.3) [*n* = 6]	47,670 (10.9) [*n* = 6]	119,600 (10.1) [*n* = 6]
AUC_0-inf_, μg∙h/mL	47,000 (28.7) [*n* = 5]	48,650 (32.9) [*n* = 6]	52,270 (17.3) [*n* = 5]	51,820 [*n* = 1]	128,700 (7.7) [*n* = 6]
C_eoi_, μg/mL	720 (16.8) [*n* = 6]	679 (18.1) [*n* = 6]	697 (19.0) [*n* = 6]	736 (8.5) [*n* = 6]	1,488 (18.1) [*n* = 6]
C_max_, μg/mL	756 (17.5) [*n* = 6]	704 (18.7) [*n* = 6]	761 (16.6) [*n* = 6]	790 (10.8) [*n* = 6]	1,547 (15.6) [*n* = 6]
T_max_, h	1.08 (1.01, 2.03) [*n* = 6]	0.55 (0.52, 0.76) [*n* = 6]	0.44 (0.34, 1.26) [*n* = 6]	1.13 (0.14, 1.13) [*n* = 6]	0.33 (0.27, 0.50) [*n* = 6]
t_1/2_, h	49.3 (9.5) [*n* = 5]	51.2 (10.3) [*n* = 6]	48.8 (1.5) [*n* = 5]	58.0 [*n* = 1]	55.0 (17.1) [*n* = 6]
CL, L/h	0.049 (30.2) [*n* = 5]	0.047 (20.2) [*n* = 6]	0.041 (4.3) [*n* = 5]	0.040 [*n* = 1]	0.034 (17.2) [*n* = 6]
Vz, L	3.42 (9.4) [*n* = 5]	3.41 (17.4) [*n* = 6]	2.88 (5.5) [*n* = 5]	3.33 [*n* = 1]	2.60 (41.6) [*n* = 6]

AUC_0-inf_, area under the serum concentration-time curve from time 0 (start of infusion) extrapolated to infinity; AUC_0-last_, area under the serum concentration-time curve from time 0 (start of infusion) to the time of the last observed nonzero concentration; C_eoi_, observed concentration at the end of infusion; CL, clearance; C_max_, maximum observed serum concentration; CV%, coefficient of variation; PK, pharmacokinetic; SD, standard deviation; t_1/2_, half-life; T_max_, time to reach maximum observed serum concentration; Vz, volume of distribution.

^a^Area under the serum concentration-time curves, C_eoi_, C_max_, CL, and Vz are presented as geometric mean and geometric CV%; T_max_ is presented as median (minimum, maximum); t_1/2_ is presented as arithmetic mean ± SD. When n = 1, geometric CV% or SD are not available.

### 3.4 PD

**Figure 3:**
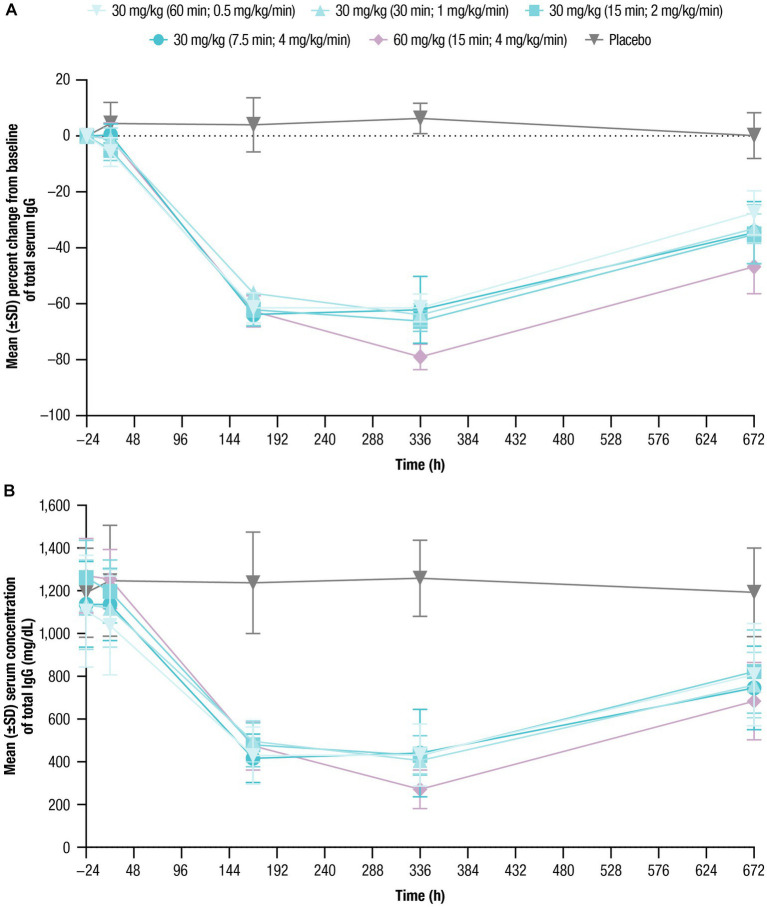
Total serum IgG concentrations over time were assessed for up to 672 h following single doses of nipocalimab (30 and 60 mg/kg) administered at escalating infusion rates (0.5–4 mg/kg/min). **(A)** Percent change from baseline of total serum IgG concentration (*n* = 6 per nipocalimab cohort, *n* = 10 for placebo cohort). **(B)** Mean (±SD) total IgG serum concentrations (*n* = 6 per nipocalimab cohort, *n* = 10 for placebo cohort). IgG, immunoglobulin G; SD, standard deviation.

Mean baseline serum IgG levels ranged from 1,105 to 1,270 mg/dL across treatment cohorts (Table 1). Following nipocalimab administration, the mean IgG values decreased from baseline compared with placebo, with similar decreases following all 30-mg/kg infusions and larger decreases following a 60-mg/kg infusion (Figure 3; Table 4). The maximum decrease from baseline in mean serum IgG levels ranged from −61.5% to −66.2% (Day 8 or Day 15) for the 30-mg/kg infusion cohorts and was 79.0% (Day 15) for the 60-mg/kg infusion cohort. The mean IgG values remained below baseline (−27.4% to −46.7% from baseline) at Day 29 for all nipocalimab dosing cohorts. The mean serum IgG values following placebo administration remained within the normal range throughout the study.

**Table 4:** Percent change from baseline in total serum IgG concentration.

Time point	Percent change from baseline in total serum IgG concentration, % (SD)					
	Nipocalimab					
	30 mg/kg (60 min; 0.5 mg/kg/min) (*n* = 6)	30 mg/kg (30 min; 1 mg/kg/min) (*n* = 6)	30 mg/kg (15 min; 2 mg/kg/min) (*n* = 6)	30 mg/kg (7.5 min; 4 mg/kg/min) (*n* = 6)	60 mg/kg (15 min; 4 mg/kg/min) (*n* = 6)	Placebo (*n* = 10)
Day 2	−5.74 (5.10)	−1.08 (3.94)	−4.95 (3.80)	0.36 (3.96)	−0.75 (5.56)	4.51 (7.57)
Day 8	−61.46 (5.38)	−56.24 (2.79)	−62.13 (5.66)	−63.82 (4.23)	−62.72 (5.63)	4.03 (9.72)
Day 15	−61.42 (4.95)	−63.92 (3.96)	−66.15 (3.67)	−62.12 (12.00)	−78.99 (4.56)	6.31 (5.46)
Day 29	−27.43 (7.87)	−33.12 (5.31)	−35.17 (10.52)	−34.50 (11.14)	−46.71 (9.64)	0.17 (8.23)^a^

IgG, immunoglobulin G; SD, standard deviation.

^a^*n* = 9.

**Figure 4:**
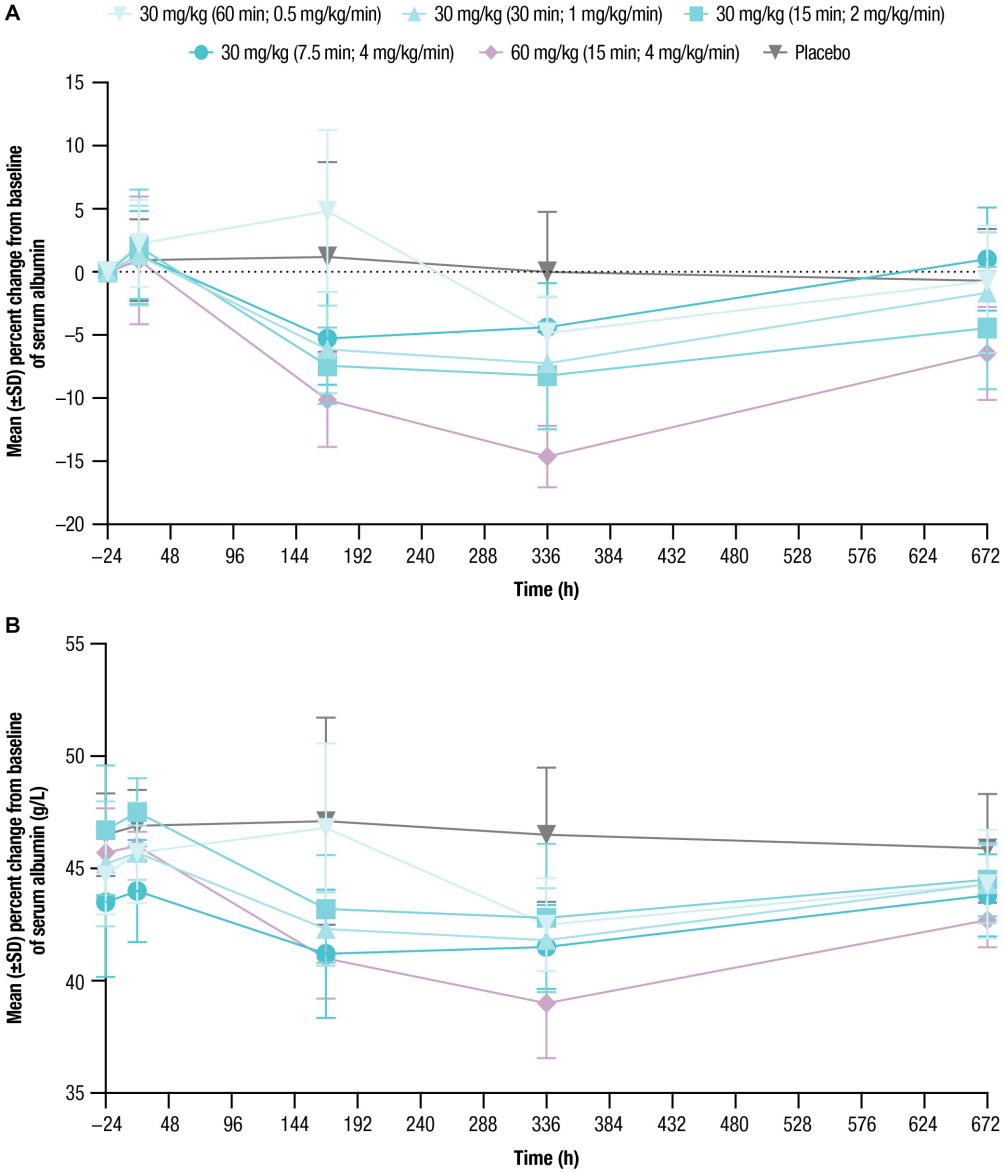
Serum albumin concentrations over time were assessed for up to 672 h following single doses of nipocalimab (30 and 60 mg/kg) administered at escalating infusion rates (0.5–4 mg/kg/min). **(A)** Percent change from baseline of serum albumin concentration (*n* = 6 per nipocalimab cohort, *n* = 10 for placebo cohort). **(B)** Mean (±SD) serum albumin concentrations (*n* = 6 per nipocalimab cohort, *n* = 10 for placebo cohort). SD, standard deviation.

Mean baseline serum albumin levels ranged from 43.5 g/L to 46.7 g/L across treatment cohorts (Table 1). Following nipocalimab administration, mean albumin values generally decreased from baseline compared with placebo. As with IgG, similar decreases followed all 30-mg/kg infusions, with larger decreases following the 60-mg/kg infusion (Figure 4; Table 5). However, the maximum decrease from baseline in mean serum albumin levels was observed in both dosing cohorts on Day 15 and ranged from −4.4% to −8.2% for the 30-mg/kg infusion cohorts and was most pronounced for the 60-mg/kg infusion cohort at −14.6%. The mean albumin values had recovered at Day 29 to near or just below baseline (−0.7% to −4.4% from baseline) in the 30-mg/kg infusion cohorts and to −6.5% in the 60-mg/kg infusion cohort. The mean serum albumin values following placebo administration remained within −1% of the normal range throughout the study. There were no clinically relevant shifts from normal at baseline to abnormal postdose in serum albumin levels in any of the nipocalimab dosing cohorts (Supplementary Table 1).

**Table 5:** Percent change from baseline in serum albumin concentration.

Time point	Percent change from baseline in serum albumin concentration, % (SD)					
	Nipocalimab					
	30 mg/kg (60 min; 0.5 mg/kg/min) (*n* = 6)	30 mg/kg (30 min; 1 mg/kg/min) (*n* = 6)	30 mg/kg (15 min; 2 mg/kg/min) (*n* = 6)	30 mg/kg (7.5 min; 4 mg/kg/min) (*n* = 6)	60 mg/kg (15 min; 4 mg/kg/min) (*n* = 6)	Placebo (*n* = 10)
Day 2	2.25 (3.45)	1.30 (3.97)	1.99 (4.52)	1.34 (3.50)	0.90 (5.07)	0.93 (3.25)
Day 8	4.80 (6.43)	−6.13 (3.49)	−7.43 (3.03)	−5.26 (3.66)	−10.15 (3.74)	1.18 (7.53)
Day 15	−4.84 (2.93)	−7.24 (5.25)	−8.22 (4.24)	−4.39 (3.49)	−14.64 (2.45)	−0.01 (4.77)
Day 29	−0.72 (4.42)	−1.66 (4.79)	−4.44 (4.86)	1.01 (4.10)	−6.47 (3.70)	−0.71 (4.12)^a^

SD, standard deviation.

^a^*n* = 9.

## 4 Discussion

This single-infusion, sequential, randomized, double-blind, placebo-controlled, escalating dose and infusion rate, phase 1 study examined the safety, tolerability, PK, and PD of nipocalimab when administered at different infusion rates and dose levels. Nipocalimab administered at doses up to 60 mg/kg and infusion rates up to 4 mg/kg/min, with infusion durations as short as 7.5 min, was well tolerated with no new safety findings and a safety profile generally comparable to placebo, highlighting the favorable safety and tolerability of nipocalimab when administered at high doses with shortened infusion duration. Nipocalimab also exhibited consistent, dose-dependent PK and induced dose-dependent total serum IgG reductions. These results provide evidence to support durations of administration as short as 7.5 min (when infusing 4 mg/kg/min with a 30-mg/kg dose). A phase 3 study of nipocalimab for gMG (ClinicalTrials.gov Identifier: NCT04951622) is underway and uses the shortened infusion durations shown in this study to be safe, with consistent PK and PD effects. The tolerability of these rapid infusion rates and the consistency of PK and PD across infusion rates and doses up to 60 mg/kg may also provide a window of safety in dosing, decreased patient burden, as well as greater ease and efficiency of administration in clinical settings.

These results support the safety of nipocalimab at higher infusion rates and at doses up to 60 mg/kg as assessed up to 28 days following single IV administration. TEAEs considered related to the study treatment occurred in both placebo and nipocalimab cohorts. There was a trend toward higher rates of the most frequent TEAEs with shorter rates of infusion. However, most of these TEAEs occurred more than 24 h after the infusion in participants receiving the highest rate of infusion. Given that the PK parameters of nipocalimab were generally similar by 24 h after the beginning of the infusion for all nipocalimab dosing cohorts regardless of infusion rate, it is unlikely that TEAEs that occurred 24 h after the infusion were infusion-rate driven. Nevertheless, it should be noted that these TEAEs could still be considered related to the study drug. No serious TEAEs or deaths occurred during the study. Altogether, these results complement the single and multiple dose safety profile observed previously with IV nipocalimab administered at 30 mg/kg weekly for up to 28 days or 30 mg/kg every 4 weeks or 60 mg/kg every other week for 57 days (Ling et al., 2019; Antozzi et al., 2024).

Overall PK profiles of nipocalimab were similar regardless of infusion rate, although a numerical trend toward shorter T_max_, lower CL, and lower Vz with higher infusion rates was observed. The PK parameters determined in this phase 1 study were generally comparable to those found in the initial phase 1 study (Ling et al., 2019).

Serum IgG reductions with nipocalimab demonstrated dose-dependence, with approximately 64% and 79% reductions from baseline following the 30-mg/kg infusions and 60-mg/kg infusion, respectively. Serum IgG reductions were not affected by infusion rates when given at a dose of 30 mg/kg, with approximately 61% and 64% maximum reductions from baseline following the 30-mg/kg infusions at the rates of 0.5 mg/kg/min and 4 mg/kg/min, respectively. The IgG reduction observed in this study are associated with the IgG levels associated with positive clinical responses observed in patients with IgG autoantibody-mediated diseases who underwent efgartigimod, PLEX or IA treatment (Liu et al., 2010; Kohler et al., 2011; Kasuya et al., 2013; VYVGART®, 2023). Serum albumin decreases observed following nipocalimab dosing remained within the normal range and within 10% of baseline for the 30-mg/kg cohorts and within 15% of baseline for the 60-mg/kg cohort. These changes were within the normal range, asymptomatic, and showed recovery toward baseline following nadir at approximately 8 to 15 days postdose as observed in the previous single dose study (Aull and McCord, 1957; Felding et al., 1980; Ling et al., 2019). These data further support the potential use of nipocalimab for treatment of IgG autoantibody-mediated diseases at durations of IV infusion as short as 7.5 min. The tolerability of nipocalimab may relate to its high affinity for the FcRn IgG binding site and aglycosylated design that contribute to its selectivity and effectorless binding (Ling et al., 2022).

The percentage of total IgG reduction in this study was similar to that observed in the previous phase 1 and Vivacity-MG studies when given at similar doses (Ling et al., 2019; Antozzi et al., 2024). By targeting IgG clearance while permitting IgG production and cellular immune functions, nipocalimab and other FcRn-targeting agents do not result in broad immunosuppression allowing sparing of corticosteroids or other immunosuppressive agents (Peter et al., 2020; Ling et al., 2022).

The limitations of this study include its small number of participants. The maximum infusion rate conducted in this study was restricted to 4 mg/kg/min as the fastest available rate of infusion at the clinical site; thus, higher infusion rates may be possible although not evaluated. Furthermore, the study included only healthy volunteers who had no significant comorbidities or concomitant medications, which does not generally reflect the impact of nipocalimab treatment on patients with IgG autoantibody-mediated disease.

The results reported here underscore the ability to safely administer nipocalimab up to 60 mg/kg with an infusion rate as high as 4 mg/kg/min in healthy individuals. These preliminary findings also support the selection of dose and infusion rate in ongoing phase 2 and 3 studies of nipocalimab for the treatment of gMG and other IgG autoantibody-mediated diseases.

## Data availability statement

The datasets presented in this article are not readily available. The data sharing policy of Janssen Pharmaceutical Companies of Johnson & Johnson is available at https://www.janssen.com/clinical-trials/transparency. As noted on this site, requests for access to the trial data can be submitted through Yale Open Data Access (YODA) Project site at http://yoda.yale.edu.

## Ethics statement

The studies involving humans were approved by Advarra, Inc. institutional review board. The studies were conducted in accordance with the local legislation and institutional requirements. The participants provided their written informed consent to participate in this study.

## Author contributions

JHL: Data curation, Formal analysis, Validation, Writing – review & editing. AV: Data curation, Formal analysis, Validation, Writing – review & editing. CA: Conceptualization, Data curation, Validation, Writing – review & editing. SA: Conceptualization, Data curation, Validation, Writing – review & editing. WSD: Conceptualization, Data curation, Validation, Writing – review & editing. LEL: Conceptualization, Data curation, Formal analysis, Validation, Writing – review & editing.

## Glossary

AE: adverse event
AUC: area under the curve
AUC_0-last_: the time of the last observed nonzero concentration
AUC_0-inf_: area under the serum concentration-time curve from time 0 (start of infusion) extrapolated to infinity
BMI: body mass index
C_eoi_: observed concentration at the end of infusion
CL: clearance
C_max_: maximum observed serum concentration
CV: coefficient of variation
ECG: electrocardiogram
EOI: end of infusion
FcRn: Fc receptor
gMG: generalized myasthenia gravis
IA: immunoadsorption
Ig: immunoglobulin
IV: intravenous
IVIG: intravenous immunoglobulin
MOI: middle of infusion
PD: pharmacodynamics
PK: pharmacokinetics
PLEX: plasma exchange
SAE: serious adverse event
SD: standard deviation
t_1/2_: half-life
TEAE: treatment-emergent adverse event
T_max_: time to reach maximum observed serum concentration
Vz: volume of distribution
