# Supplementary material for: Pharmacokinetics and pharmacodynamics across infusion rates of intravenously administered nipocalimab: results of a phase 1, placebo-controlled study
Source: Front Neurosci. 2024 Feb 1;18:1302714. doi: 10.3389/fnins.2024.1302714 (PMC10867144; doi:10.3389/fnins.2024.1302714)
Supplement: Supplementary file 1 [file Table_1.DOCX]

# *Supplementary Material*

# Supplementary Table 1. Proportion (%) of Participants with Grades 1, 2, or 3 Hypoalbuminemia; Normal; or High Serum Albumin Concentration at Postdose Versus at Baseline

| Cohort | Time point | Participants within a category at postdose, n/N (%) | Participants within a category at baseline, n/N (%) | | | | |
| --- | --- | --- | --- | --- | --- | --- | --- |
|  |  |  | **Grade 1** | **Grade 1** | **Grade 1** | **Grade 1** | **Grade 1** |
| **30 mg/kg**  **(60 min; 0.5 mg/kg/min)** | **Day 2** | **Grade 3**  **Grade 2**  **Grade 1**  **Normal**  **High** | 0  0  0  0  0 | 0  0  0  0  0 | 0  0  0  0  0 | 0  0  0  6/6 (100)  0 | 0  0  0  0  0 |
|  | **Day 8** | **Grade 3**  **Grade 2**  **Grade 1**  **Normal**  **High** | 0  0  0  0  0 | 0  0  0  0  0 | 0  0  0  0  0 | 0  0  4/6 (66.7)  2/6 (33.3)  0 | 0  0  0  0  0 |
|  | **Day 15** | **Grade 3**  **Grade 2**  **Grade 1**  **Normal**  **High** | 0  0  0  0  0 | 0  0  0  0  0 | 0  0  0  0  0 | 0  0  0  6/6 (100)  0 | 0  0  0  0  0 |
|  | **Day 29** | **Grade 3**  **Grade 2**  **Grade 1**  **Normal**  **High** | 0  0  0  0  0 | 0  0  0  0  0 | 0  0  0  0  0 | 0  0  0  6/6 (100)  0 | 0  0  0  0  0 |

| Cohort | Time point | Participants within a category at postdose, n/N (%) | Participants within a category at baseline, n/N (%) | | | | |
| --- | --- | --- | --- | --- | --- | --- | --- |
|  |  |  | **Grade 1** | **Grade 2** | **Grade 1** | **Normal** | **Grade 1** |
| **30 mg/kg**  **(30 min; 1 mg/kg/min)** | **Day 2** | **Grade 3**  **Grade 2**  **Grade 1**  **Normal**  **High** | 0  0  0  0  0 | 0  0  0  0  0 | 0  0  0  0  0 | 0  0  0  6/6 (100)  0 | 0  0  0  0  0 |
|  | **Day 8** | **Grade 3**  **Grade 2**  **Grade 1**  **Normal**  **High** | 0  0  0  0  0 | 0  0  0  0  0 | 0  0  0  0  0 | 0  0  0  6/6 (100)  0 | 0  0  0  0  0 |
|  | **Day 15** | **Grade 3**  **Grade 2**  **Grade 1**  **Normal**  **High** | 0  0  0  0  0 | 0  0  0  0  0 | 0  0  0  0  0 | 0  0  1/6 (16.7)  5/6 (83.3)  0 | 0  0  0  0  0 |
|  | **Day 29** | **Grade 3**  **Grade 2**  **Grade 1**  **Normal**  **High** | 0  0  0  0  0 | 0  0  0  0  0 | 0  0  0  0  0 | 0  0  0  6/6 (100)  0 | 0  0  0  0  0 |

| Cohort | Time point | Participants within a category at postdose, n/N (%) | Participants within a category at baseline, n/N (%) | | | | |
| --- | --- | --- | --- | --- | --- | --- | --- |
|  |  |  | **Grade 1** | **Grade 2** | **Grade 1** | **Normal** | **Grade 1** |
| **30 mg/kg**  **(15 min; 2 mg/kg/min)** | **Day 2** | **Grade 3**  **Grade 2**  **Grade 1**  **Normal**  **High** | 0  0  0  0  0 | 0  0  0  0  0 | 0  0  0  0  0 | 0  0  0  5/6 (83.3)  0 | 0  0  0  1/6 (16.7)  0 |
|  | **Day 8** | **Grade 3**  **Grade 2**  **Grade 1**  **Normal**  **High** | 0  0  0  0  0 | 0  0  0  0  0 | 0  0  0  0  0 | 0  0  0  5/6 (83.3)  0 | 0  0  0  1/6 (16.7)  0 |
|  | **Day 15** | **Grade 3**  **Grade 2**  **Grade 1**  **Normal**  **High** | 0  0  0  0  0 | 0  0  0  0  0 | 0  0  0  0  0 | 0  0  1/6 (16.7)  4/6 (66.7)  0 | 0  0  0  1/6 (16.7)  0 |
|  | **Day 29** | **Grade 3**  **Grade 2**  **Grade 1**  **Normal**  **High** | 0  0  0  0  0 | 0  0  0  0  0 | 0  0  0  0  0 | 0  0  0  5/6 (83.3)  0 | 0  0  0  1/6 (16.7)  0 |

| Cohort | Time point | Participants within a category at postdose, n/N (%) | Participants within a category at baseline, n/N (%) | | | | |
| --- | --- | --- | --- | --- | --- | --- | --- |
|  |  |  | **Grade 1** | **Grade 2** | **Grade 1** | **Normal** | **Grade 1** |
| **30 mg/kg**  **(7.5 min; 4 mg/kg/min)** | **Day 2** | **Grade 3**  **Grade 2**  **Grade 1**  **Normal**  **High** | 0  0  0  0  0 | 0  0  0  0  0 | 0  0  0  0  0 | 0  0  0  6/6 (100)  0 | 0  0  0  0  0 |
|  | **Day 8** | **Grade 3**  **Grade 2**  **Grade 1**  **Normal**  **High** | 0  0  0  0  0 | 0  0  0  0  0 | 0  0  0  0  0 | 0  0  3/6 (50.0)  3/6 (50.0)  0 | 0  0  0  0  0 |
|  | **Day 15** | **Grade 3**  **Grade 2**  **Grade 1**  **Normal**  **High** | 0  0  0  0  0 | 0  0  0  0  0 | 0  0  0  0  0 | 0  0  1/6 (16.7)  5/6 (83.3)  0 | 0  0  0  0  0 |
|  | **Day 29** | **Grade 3**  **Grade 2**  **Grade 1**  **Normal**  **High** | 0  0  0  0  0 | 0  0  0  0  0 | 0  0  0  0  0 | 0  0  0  6/6 (100)  0 | 0  0  0  0  0 |

| Cohort | Time point | Participants within a category at postdose, n/N (%) | Participants within a category at baseline, n/N (%) | | | | |
| --- | --- | --- | --- | --- | --- | --- | --- |
|  |  |  | **Grade 1** | **Grade 2** | **Grade 1** | **Normal** | **Grade 1** |
| **60 mg/kg**  **(15 min; 4 mg/kg/min)** | **Day 2** | **Grade 3**  **Grade 2**  **Grade 1**  **Normal**  **High** | 0  0  0  0  0 | 0  0  0  0  0 | 0  0  0  0  0 | 0  0  0  6/6 (100)  0 | 0  0  0  0  0 |
|  | **Day 8** | **Grade 3**  **Grade 2**  **Grade 1**  **Normal**  **High** | 0  0  0  0  0 | 0  0  0  0  0 | 0  0  0  0  0 | 0  0  1/6 (16.7)  5/6 (83.3)  0 | 0  0  0  0  0 |
|  | **Day 15** | **Grade 3**  **Grade 2**  **Grade 1**  **Normal**  **High** | 0  0  0  0  0 | 0  0  0  0  0 | 0  0  0  0  0 | 0  0  3/6 (50.0)  3/6 (50.0)  0 | 0  0  0  0  0 |
|  | **Day 29** | **Grade 3**  **Grade 2**  **Grade 1**  **Normal**  **High** | 0  0  0  0  0 | 0  0  0  0  0 | 0  0  0  0  0 | 0  0  0  6/6 (100)  0 | 0  0  0  0  0 |

| Cohort | Time point | Participants within a category at postdose, n/N (%) | Participants within a category at baseline, n/N (%) | | | | |
| --- | --- | --- | --- | --- | --- | --- | --- |
|  |  |  | **Grade 1** | **Grade 2** | **Grade 1** | **Normal** | **Grade 1** |
| **Placebo** | **Day 2** | **Grade 3**  **Grade 2**  **Grade 1**  **Normal**  **High** | 0  0  0  0  0 | 0  0  0  0  0 | 0  0  0  0  0 | 0  0  0  10/10 (100)  0 | 0  0  0  1/6  0 |
|  | **Day 8** | **Grade 3**  **Grade 2**  **Grade 1**  **Normal**  **High** | 0  0  0  0  0 | 0  0  0  0  0 | 0  0  0  0  0 | 0  0  0  8/10 (80.0)  2/10 (20.0) | 0  0  0  0  0 |
|  | **Day 15** | **Grade 3**  **Grade 2**  **Grade 1**  **Normal**  **High** | 0  0  0  0  0 | 0  0  0  0  0 | 0  0  0  0  0 | 0  0  0  9/10 (90.0)  1/10 (10.0) | 0  0  0  0  0 |
|  | **Day 29** | **Grade 3**  **Grade 2**  **Grade 1**  **Normal**  **High** | 0  0  0  0  0 | 0  0  0  0  0 | 0  0  0  0  0 | 0  0  0  8/9 (88.9)  1/9 (11.1) | 0  0  0  0  0 |

N, number of participants dosed assessed at the time point.
